# Mechanistic Insight into Binding of Huperzine A with Human Serum Albumin: Computational and Spectroscopic Approaches

**DOI:** 10.3390/molecules27030797

**Published:** 2022-01-25

**Authors:** Anas Shamsi, Moyad Shahwan, Mohd Shahnawaz Khan, Fahad A. Alhumaydhi, Suliman A. Alsagaby, Waleed Al Abdulmonem, Bekhzod Abdullaev, Dharmendra Kumar Yadav

**Affiliations:** 1 Centre for Interdisciplinary Research in Basic Sciences, Jamia Millia Islamia, Jamia Nagar, New Delhi 110025, India; 2 Centre of Medical and Bio-Allied Health Sciences Research, Ajman University, Ajman P.O. Box 346, United Arab Emirates; moyad76@hotmail.com; 3 College of Pharmacy & Health Sciences, Ajman University, Ajman P.O. Box 346, United Arab Emirates; 4 Department of Biochemistry, College of Sciences, King Saud University, Riyadh 11451, Saudi Arabia; moskhan@ksu.edu.sa; 5 Department of Medical Laboratories, College of Applied Medical Sciences, Qassim University, Buraidah 52571, Saudi Arabia; f.alhumaydhi@qu.edu.sa; 6 Department of Medical Laboratory Sciences, College of Applied Medical Sciences, Majmaah University, Majmaah 11932, Saudi Arabia; s.alsaqaby@mu.edu.sa; 7 Department of Pathology, College of Medicine, Qassim University, Buraidah 51452, Saudi Arabia; waleedmonem@qumed.edu.sa; 8 Scientific Department, Akfa University, Tashkent 100022, Uzbekistan; b.abdullaev@akfauniversity.org; 9 College of Pharmacy, Gachon University of Medicine and Science, Hambakmoeiro, Yeonsu-gu, Incheon 21924, Korea

**Keywords:** Huperzine A, molecular dynamics simulation, fluorescence spectroscopy, human serum albumin, neurodegenerative disorders, drug–protein interactions

## Abstract

Human serum albumin (HSA) is the most abundant protein in plasma synthesized by the liver and the main modulator of fluid distribution between body compartments. It has an amazing capacity to bind with multiple ligands, offering a store and transporter for various endogenous and exogenous compounds. Huperzine A (HpzA) is a natural sesquiterpene alkaloid found in *Huperzia serrata* and used in various neurological conditions, including Alzheimer’s disease (AD). This study elucidated the binding of HpzA with HSA using advanced computational approaches such as molecular docking and molecular dynamic (MD) simulation followed by fluorescence-based binding assays. The molecular docking result showed plausible interaction between HpzA and HSA. The MD simulation and principal component analysis (PCA) results supported the stable interactions of the protein–ligand complex. The fluorescence assay further validated the in silico study, revealing significant binding affinity between HpzA and HSA. This study advocated that HpzA acts as a latent HSA binding partner, which may be investigated further in AD therapy in experimental settings.

## 1. Introduction

HSA is a major transporter and the most abundant protein in the plasma. It is responsible for balancing the osmotic pressure and is a major regulator of fluid distribution in the body. HSA exhibits astonishing ligand-binding capabilities, acting as a warehouse and transporter of many endo- and exogenous compounds [1]. This promiscuous, nonspecific affinity can lead to sudden changes in concentrations caused by displacement when two or more compounds compete for binding to the same molecular site [2]. HSA, a major carrier in the human circulatory system performs a key function of circulating various compounds such as drugs [3]. HSA interactions with drugs alter proteins’ pharmacokinetics and pharmacodynamics as well. Free drugs diffuse inactively across the membranes via specific transporters to interact with their respective protein targets [4]. A pivotal step in the domain of drug discovery is the investigation of the pharmacokinetics and pharmacodynamics of drugs. Pharmacological profiling of drugs offers understanding of the interactions of vital therapeutic drugs or derivatives with either plasma or target tissue proteins [5]. The binding of a drug to HSA is a critical factor determining its pharmacological profiling and distribution [6]. In medicinal chemistry, studies pertaining to plasma proteins and drugs binding are attracting researchers across the globe, because these studies provide a platform to study drugs’ behavior and action, thereby delineating their transport and distribution characteristics in the circulatory system. Furthermore, it is also vital to study the protein–protein or protein–drug interactions to make progressive inroads in the advancements made in pharmaceutical industry [7]. HSA is the main plasma protein, and it is imperative to study binding of drugs with HSA. Thus, this study aimed at investigating the binding mechanism of HpzA to HSA. HSA is a one-chained polypeptide weighing 66.5 kDa. The 585 amino acid polypeptide assembles in a heart-shaped structure [1]. The protein displays two specific sites for drug binding as specified using Sudlow’s fluorescent probe displacement method. According to the Sudlow’s classification, there are two main binding sites for drug ligands of HSA, namely, subdomain IIA (site IIA) and IIIA (site IIIA) [8]. Site IIA, positioned in subdomain IIA, concerns with the selective binding of heterocyclic anions, while aromatic carboxylates bind to the site IIIA that is located in subdomain IIIA,. For example, ibuprofen binds to site IIIA and warfarin to site IIA [9]. The binding of all the ligands cannot be considered under Sudlow’s model. Site IB, a third binding pocket, located in subdomain IB, is a hydrophobic D-shaped cavity, is also used for interaction by some compounds such as bilirubin [10]. A study [11] suggested site IB as the third major site having the potential to bind drug ligands of HSA.

AD, the sixth leading cause of claiming lives worldwide, is a progressive, neurodegenerative disorder. The condition is characterized by impairments in mental functions, amnesia, and dementia [12]. The major hallmarks of the condition are accumulated amyloid β-peptide (Aβ) in plaques, the formation of neurofibrillary tangles (NFTs), and degeneration of neurons. The mechanism by which neurons degenerate is still unclear and needs extensive research. Aβ accumulation and deposition can cause neurodegeneration by many mechanisms, such as inflammation, oxidative stress, and apoptosis. In addition to the genetic factors, environmental factors such as neurotoxins, stress, etc. play a major role in AD progression [13]. Reactive oxygen species (ROS) creates a condition of oxidative stress that leads to cell death, oxidative bursts, accumulation of spare free metals, etc. These have been hypothesized as major mediators in AD progression and neurodegeneration. Postmortem reports have indicated apoptosis as a major event in neurodegeneration and AD [14].

HpzA, a Lycopodium alkaloid, is a Chinese medicine isolated from *Qiang Ceng Ta*, the whole plant of *Huperzia serrata*. The plant belongs to the Huperziaceae family, and it has gained wide popularity due to its anticholinesterase (AChE) and anti-AD properties. The alkaloid also acts as a remedy for various ailments such as strains and swellings and neurological disorders such as schizophrenia and neurodegenerative disorders [15]. HpzA, a well-known AChE inhibitor, has properties superior to those of FDA-approved drugs such as donepezil, galantamine, etc. [16]. Figure S1 describes the 2D and 3D structural features of HpzA. Extensive clinical trials for the HpzA conducted in China showed enhanced memory in elderly individuals, subjects with issues related to amnesia, and AD patients. The naturally extracted ligand has also been through various extensive clinical trials on vascular dementia, showing improved cognitive function in subjects with AD [17]. In this introductory study, we report the plausible binding of HpzA with HSA. A molecular docking study was performed primarily to explore the possible interactions and binding affinity of HpzA towards HSA and was followed by MD simulation studies for 100 ns. Computational studies suggested plausible binding of HpzA with HSA, which was further validated by fluorescence-based binding assays. Thus, this study delineates the mechanism of binding of HpzA with HSA by employing spectroscopic and in silico approaches.

## 2. Results

### 2.1. Molecular Docking

**Figure 1:**
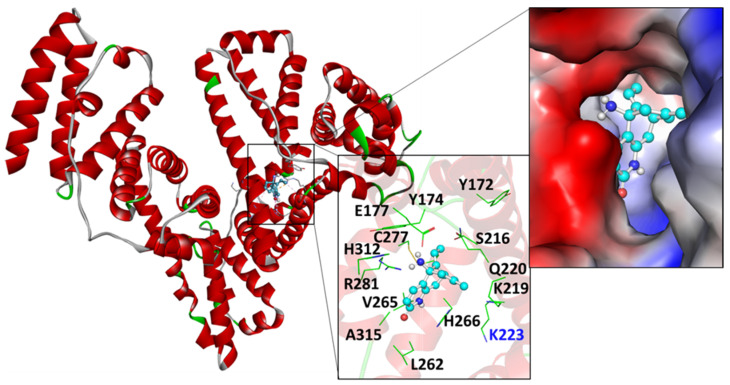
The binding of HpzA with HSA. Ribbon and surface representation of HSA protein showing various interactions and docking fit of HpzA (UniProt ID: P02768).

The functional activity of a protein changes broadly upon conformational alterations induced by ligand binding. The molecular docking approach can systematically consider potential binding modes for a protein–ligand system [18]. The blind docking approach was used to find all possible interactions between HpzA and HSA. HpzA has multiple binding sites on has, with varying affinities. All the docking sites and their docking energies are depicted in Figures S2 and S3, respectively. Out of nine docked conformations of HpzA in the splitting process, the preferable docked conformation was taken and explored in detail. The selected conformation of the docked HpzA showed a considerable binding score of −7.2 kcal/mol. It had LE and pKi values 0.4 (kcal/mol/non-H atom) and 5.28, respectively. The docking score affirmed HpzA as a plausible binding partner of HSA. Figure 1 shows the binding mode of HpzA with HSA. HpzA was shown to have a decent structural complementarity with good binding affinity. It showed various interactions with the key residues of the HSA binding pocket, such as one hydrogen bond with His266 and a few other noncovalent interactions with important HSA residues (Figure 1). The binding site of HpzA is located in domain II of HSA, but Tyr172, Tyr174, and Glu177 of domain I also participate in the interaction. HpzA binds at the Sudlow site I of HSA. The surface representation of HSA showed HpzA fitted inside the deep cavity of the binding site, which might cause significant changes to the conformational activity of HSA (Figure 1).

**Figure 2:**
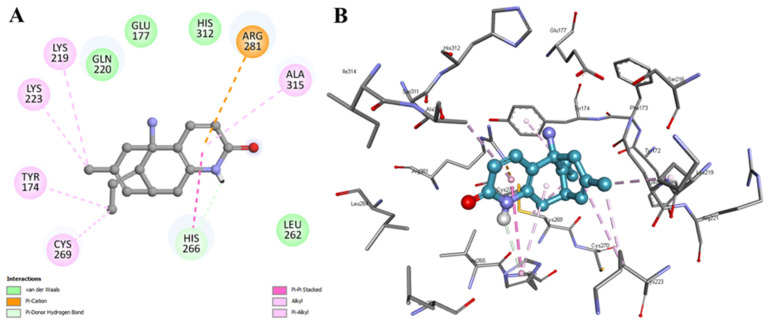
Detailed interactions of HSA with HpzA as (**A**) 2D and (**B**) 3D plots.

The selected docking pose of HpzA with HSA was further subjected to a detailed analysis of possible interactions. The generated 2D plot showed that HpzA occupied the hydrophobic pocket of HSA bordered by residues Tyr174, Glu177, Lys219, Gln220, Lys223, Leu262, His266, Cys269, Arg281, His312, and Ala315 (Figure 2A). The plot suggested that HpzA was tightly bound with HSA with the help of one hydrogen bond with His266 and several hydrophobic contacts. HpzA showed multiple van der Waals interactions with Glu177, Gln220, Leu262, and His312 of HSA. The phenolic moiety at the end of HpzA occupied a crucial binding cleft of HSA formed by different residues (Figure 2B). As a whole, the HpzA binding with HSA suggested that it could disrupt the structural conformation of the protein, which may further result in altering HSA’s binding capability.

### 2.2. MD Simulations

**Figure 3:**
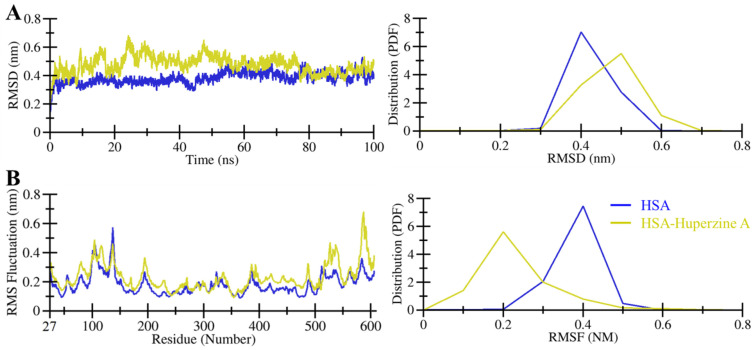
Dynamics performance of HSA. (**A**) RMSD plot of HSA and its docked complex with HpzA. (**B**) Residual fluctuation plot of HSA and its docked complex with HpzA.

Many studies have used the MD simulation approach to investigate ligand binding with proteins [7,19,20,21]. All-atom MD simulations were carried out for the docked complex of HSA–HpzA and compared with those for the free state of HSA. Plots for root mean square deviation (RMSD), root mean square fluctuation (RMSF), radius of gyration (*R*_g_), and solvent accessible surface area (SASA) fluctuations, along with hydrogen bond analysis, were generated and analyzed in detail. The analysis of RMSD assists in understanding the stability of a protein and protein–ligand complex. To explore structural deviations in HSA, we used RMSD examination of the simulated protein and protein–ligand complex. As examined, both HSA and the HSA–HpzA complex reached the equilibrium phase without any major shift in the RMSD pattern (Figure 3A, left panel). The protein–ligand complex was stable throughout 100 ns simulation. The analysis of the generated plots suggested a small amount of fluctuation at the beginning in the RMSD values of the HSA–HpzA that was possibly due to the initial adjustment of the system. As a whole, HSA showed a small number of fluctuations in its backbone in the simulation, but no significant shift was observed (Figure 3A, right panel).

Exploring RMSFs is helpful in understanding the fluctuations in the protein residues during the course of the simulation [22,23]. The RMSFs were recorded for each residue in HSA and plotted for analysis purposes. The RMSF analysis emphasized possible residual movements in HSA in free and ligand-bound states (Figure 3B). The compared RMSF values between the free and ligand-bound HSA suggested that the residual fluctuations were decreased and compacted the ligand-bound state of HSA during the simulation (Figure 3B, left panel). The results suggested that the fluctuations in the residues were minimized upon binding of HpzA. However, a few increased fluctuations were observed in the HSA residues corresponding to the loop region far from the ligand site. RMSF values distribution plot revealed that the residual fluctuations were minimized after HpzA binding, which further confirmed a robust constancy of the protein–ligand complex (Figure 3B, right panel).

**Figure 4:**
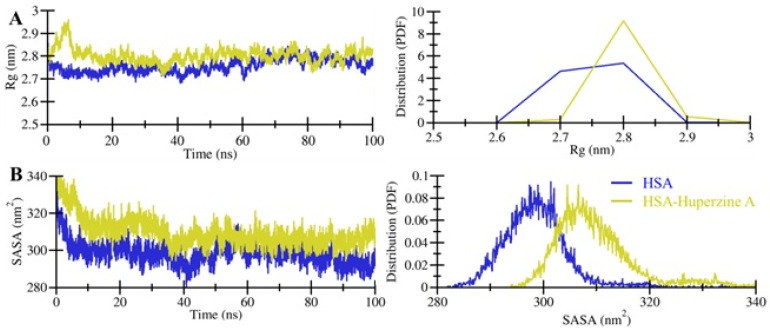
Structural compactness of HSA as a function of time. (**A**) Time evolution of the radius of gyration. (**B**) SASA plot of HSA as a function of time.

Calculating and examining the *R_g_* of a protein is useful to measure the shape of the protein based on its hydrodynamic radius [18,23,24,25]. The *R_g_*s of HSA and the HSA–HpzA docked complex were calculated to assess their structural compactness during the simulation (Figure 4A). The average *R_g_* values of free HSA and the HSA–HpzA docked complex were 2.76 nm and 2.80 nm, respectively. The pattern of *R_g_* values of HSA was like that of the *R_g_* values of HSA and the HSA–HpzA docked complex, but with a minor increase (Figure 4A, left panel). This minor increase could have resulted from the occupancy of intramolecular space of HSA by HpzA. The distribution plot of *R_g_* values also indicated a slight increase in the *R_g_* of the HSA–HpzA docked complex compared to that of the free state of HSA (Figure 4A, right panel).

SASA delineates the folding mechanism of a protein structure by analyzing the protein’s solvent accessibility [26]. SASA values were calculated and plotted to investigate the surface area of HSA and the HSA–HpzA docked complex during the simulation, the (Figure 4B). The results indicated that the SASA values were minorly increased for HSA in ligand-bound state. This slight increase in the values of SASA indicated that a number of internal residues might be exposed to the surface post the binding of HpzA (Figure 4B, left panel). The distribution plot of SASA values also indicated a slight increase in SASA of the HSA–HpzA docked complex (Figure 4B, right panel).

#### Dynamics of Intra-/Intermolecular Hydrogen Bonding

**Figure 5:**
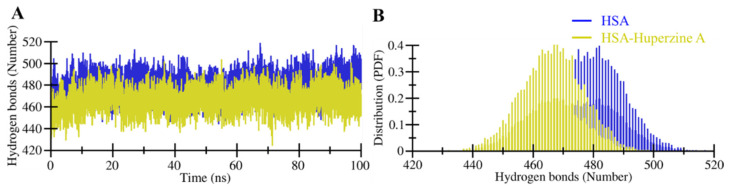
Intramolecular hydrogen bonds during the simulation. (**A**) Time evolution and stability of hydrogen bonds formed intramolecular within HSA. (**B**) The probability of distribution of hydrogen bonding as PDF.

Hydrogen bonds are necessary for maintaining the structural conformation of a protein [27]. Analysis of hydrogen bonding is useful to assess the stability of a protein and protein–ligand complex [27]. The stability of HSA and the HSA–HpzA docked complex was examined by exploring the formation of intramolecular hydrogen bonds (Figure 5). The analysis of the intramolecular hydrogen bonds formed within HSA indicated the formation of several hydrogen bonds, which maintained the integrity of HSA’s three-dimensional structure. The average hydrogen bonds estimated within HSA before and after the HpzA binding were 480 and 467, respectively. The plot indicated a significant decline in hydrogen bonds in HSA when it formed a complex with HpzA, which was possibly due to the ligand’s occupancy of intramolecular space in the binding pocket of HSA. The binding of HpzA might cause a hindrance in the formation of intramolecular hydrogen bonding within HSA. However, the plot suggested that this decrement in the formation did not lead to any major shift, which resulted in maintaining the geometry of the HSA structure during the simulation. The PDF distribution also indicated a decrease in intramolecular hydrogen bonding within HSA when bound to HpzA (Figure 5B).

**Figure 6:**
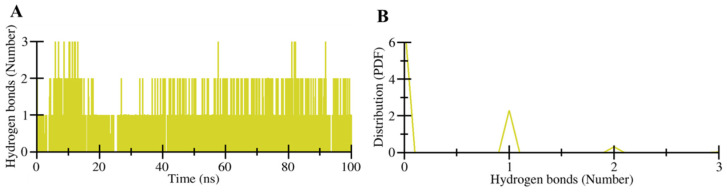
Intermolecular hydrogen bonds during the simulation. (**A**) Time evolution of hydrogen bonds formed between HpzA and HSA. (**B**) The probability of distribution of hydrogen bonds.

The formation of intermolecular hydrogen bonds plays a crucial role in the stability and directionality of a protein–ligand complex [28]. The intermolecular hydrogen bonds can be explored to measure the strength of a ligand towards the binding pocket of a protein. The hydrogen bonds formed between HpzA and HSA were recorded and plotted to explore their formation and breakdown during the simulation (Figure 6A). Analysis of the generated plot indicated the formation of up to three hydrogen bonds during the simulation time. The analysis clearly indicated that at least one hydrogen bond was formed during the simulation time. This strong hydrogen bond could be correlated with the molecular docking observation that His266 was involved in the conventional hydrogen bond formation. The analysis suggested the importance of intermolecular hydrogen bonding in the ligand binding to form a stable complex. The PDF plot indicated that at least one hydrogen bond was formed with higher probability and stability in the HSA–HpzA docked complex (Figure 6B).

### 2.3. Principal Component and Free Energy Landscape Analyses

**Figure 7:**
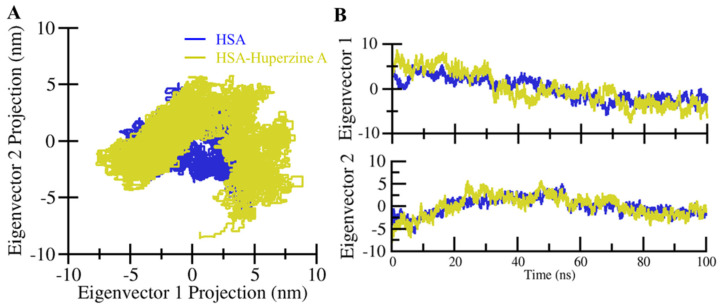
Principal component analysis. **(A)** Two-dimensional projections of HSA trajectories on eigenvector 1 and eigenvector 2. (**B**) Time evaluation of conformational projections of trajectories on eigenvector 1 and eigenvector 2.

Principal component analysis (PCA) is an arithmetic technique to reduce the complexity of MD trajectories by obtaining the collective motion of Cα atoms. It is vital to evaluate the stability of proteins and protein–ligand complexes [29,30]. PCA was performed to evaluate the conformational projection and transition dynamics of HSA with HpzA. The 2D projections of the first two principal components (PCs), PC1 and PC2, for HSA and HSA–HpzA is presented in Figure 7A. The analysis revealed that the 2D projection of HSA showed smaller phase space than that of HSA–HpzA. The projection of the first two eigenvectors (EVs), EV1 and EV2, showed the time evolution of the HSA projection, which indicated a similar pattern of trajectories (Figure 7B). The PCA showed that HSA and HSA–HpzA had similar correlated motions without significant change. However, the ligand-bound HSA explored a wider phase space on both EVs, which indicated its higher dynamics. The PCA confirmed that the HSA–HpzA complex was quite stable during the simulation and behaved like the free state of HSA.

**Figure 8:**
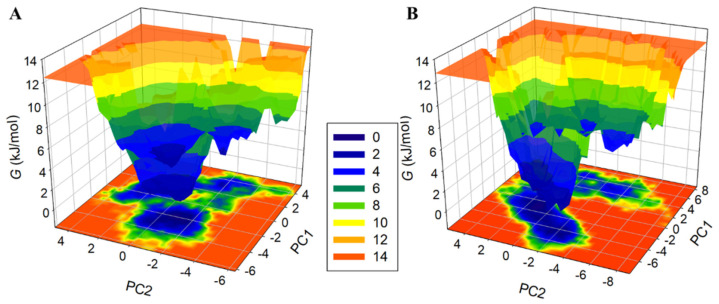
The Gibbs free energy landscapes of (**A**) free HSA and (**B**) HSA–HpzA obtained during 100 ns MD simulation.

Free energy landscape (FEL) analysis based on PCA has been utilized in representing protein conformations in a time evolution manner [31]. The FEL distinguishes the kinetic and thermodynamic estates of proteins and protein–ligand complexes. The FELs depend on the prospect of an arrangement of specific data points and converting them to free-energy values. FELs analysis was performed to investigate the conformational stability underlying HSA–HpzA binding. Figure 8 shows the FEL plots projected onto PC1 and PC2 of HSA and the HSA–HpzA complex for Cα atoms. The centralized blue zones suggested that the subsequent system was stable at that moment. The dimension and appearance of the minimum energy space, called the global minimum (in blue) in the FELs, indicated that the HSA–HpzA system was more stable than HSA. HSA and HSA–HpzA displayed a single global minimum confined within a large basin. The FEL analysis further validated our previous observations that HSA, when bound with HpzA, was stable and reached a more thermodynamically stable conformational state (Figure 8B).

### 2.4. Fluorescence-Based Binding

**Figure 9:**
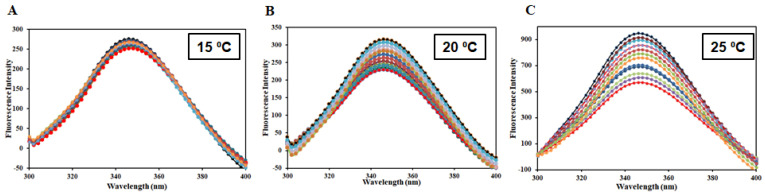
Fluorescence emission spectra of HSA in the absence and presence of varying HpzA concentrations (0–11 μM) at (**A**) 15 °C, (**B**) 20 °C, and (**C**) 25 °C.

**Figure 10:**
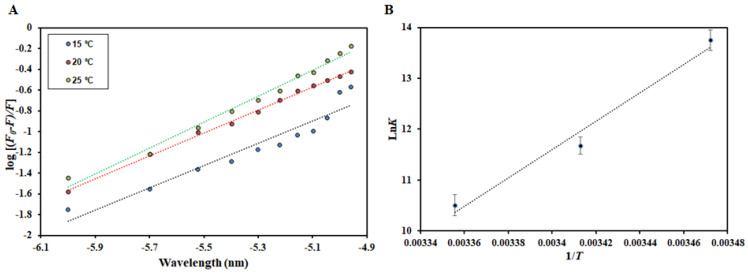
(**A**) Modified Stern–Volmer plot of the HSA–HpzA complex at different temperatures. (**B**) van ‘t Hoff plot of the HSA–HpzA complex.

Fluorescence spectroscopy serves as an important technique to provide an insight into the protein–ligand interactions, revealing different binding parameters that give an idea about the strength of interaction between a protein and ligand [32]. Intrinsic fluorescence depicts changes in the local microenvironment of aromatic amino acid residues and this serves as an important technique in determining the protein-ligand complex formation [33,34]. When a decrease in the fluorescence of native protein is observed with increasing concentration of the ligands, it is referred to as fluorescence quenching [35]. Fluorescence quenching was performed at three different temperatures (15, 20, and 25 °C). Fluorescence emission spectra of free HSA and HSA with different HpzA concentrations (0–11 µM) at various temperatures are shown in Figure 9A–C. Native HSA showed fluorescence emission maxima at 346 nm. There was a noticeable decrease HSA’s fluorescence intensity with increasing HpzA concentration, with no peak shift. Fluorescence quenching can be either static, dynamic, or a combination of both [36]. The deviation of the binding parameters with temperature delineates the operative quenching for a particular interaction. The quenching data obtained were fitted into the Stern–Volmer, modified Stern–Volmer, and van ‘t Hoff equations to obtain various binding and thermodynamic parameters for HSA–HpzA interaction as per previously published reports [37,38]. Table 1 shows the obtained values of the Stern–Volmer constant (*K*_sv_) at various temperatures, which were found to increase with increasing temperature, revealing the existence of the dynamic mode. Moreover, to confirm the quenching mode, a biomolecular quenching rate constant (*K*_q_) was calculated using *K*_sv_ = *K*_q_/τ_0_ (τ_0_ = 2.7 × 10^−9^ s), and the value was found to be higher than that of the maximum dynamic quenching constant (nearly 10^10^ M^−1^ s^−1^) [39], confirming the existence of a combination of static and dynamic quenching. Figure 10A shows the experimental fitting obtained in accordance with the modified Stern–Volmer equation. The slope of the plot gives the number of binding sites (*n*) while the intercept gives the binding constant (*K*). HpzA binds to HSA with a high binding affinity, (*K* = 9.3 × 10^5^ M^−1^ at 25 °C). The value of *K* was found to increase at a higher temperature suggesting that the HSA–HpzA complex is more stable at high temperatures. These observations affirm in silico results advocating the significant binding affinity between HpzA and HSA. Thermodynamic parameters were also found for the HSA–HpzA complex fitting the obtained data in the van ‘t Hoff equation. Figure 10B shows the van ‘t Hoff plot obtained for the HSA–HpzA complex. Table 1 shows the obtained thermodynamic parameters for the complex. Negative Δ*H* and Δ*S* implied the van der Waals and hydrogen bonding as the dominant forces driving the complex formation. Moreover, a negative Δ*G* implied the spontaneous nature of the reaction.

## 3. Materials and Methods

### 3.1. Materials

HSA (fatty acid-free) and HpzA (42643) were purchased from Sigma-Aldrich Co. (St. Louis, MO, USA). We used double distilled for the preparation of all buffers. HSA stock solution (75 μM) was made in 20 mM sodium phosphate buffer, pH 7.4. Appropriate blanks were used as control, and the reported spectra are the subtracted spectra. We used analytical grade chemicals for buffer preparations.

### 3.2. Receptor and Ligand Preparation

HSA structure was obtained from the Protein Data Bank with PDB-ID: 6HSC in a three-dimensional state. The structure of HpzA was taken in SDF format from the PubChem database with PubChem CID: 854026 in a three-dimensional state. The water molecules and other cocrystallized ligands (i.e., aristolochic acid, myristic acid, and 1,2-ethanediol) present in the protein structure were removed PDB file and optimized through the Swiss-PDB-Viewer tool (2021) [40]. The PDB files of receptor HSA and SDF file of the ligand HpzA were converted in the required format, i.e., PDBQT by InstaDock [41]. Appropriate atom types were assigned to both protein and ligand structures before performing docking.

### 3.3. Molecular Docking

The prepared files of the receptor and ligand structures were utilized for docking study using the InstaDock software [41]. InstaDock was used to calculate binding free energy between the protein–ligand complex by employing the AutoDock Vina [42] scoring function. The grid box for docking search was set blindly to facilitate free moving and searching on HSA by HpzA. The grid coordinates were summarized as 86 Å × 62 Å × 76 Å, centralized at 25.53, 9.51, and 20.27 for X, Y, and Z, respectively. The spacing of 1 Å was utilized in the docking box with default parameters. The docking result was saved in a separate directory for further analysis of the protein–ligand complex. The negative decimal logarithms of the inhibition constant as pKi and ligand efficiency were also estimated from the docking result through the inbuilt program of InstaDock.

### 3.4. Interaction Analysis

To explore the possible interactions between HpzA and HSA, all the docked conformations for the ligand output file were separated. The interaction analysis of HpzA with HSA was carried out through the Discovery Studio Visualizer (2021) [43] and PyMOL (2021) [44] tools. First, the binding pose of HpzA with HSA was selected based on the specific interaction, and then the HSA–HpzA protein–ligand complex was generated for further studies in MD simulation. 

### 3.5. MD Simulations

GROMACS [45] v.2020 beta was utilized to execute the all-atom MD simulations for examining the reliability of the HSA–HpzA docking complex in solvent conditions. The Gromacs parameters for HpzA were generated from the PRODRG server [46] to prepare a protein–ligand complex system. Free HSA and the docked complex were implanted in the simple point charge (SPC) solvent model. HSA and the docked complex, HSA–HpzA, were neutralized with an appropriate number of counter ions (Na^+^ and Cl^−^). The salt concentration was kept at physiological condition, i.e., 0.15 M. Both systems were stipulated on periodic boundary conditions, and the SHAKE algorithm was applied for limiting the movement of all bonds. The energy minimization of both the systems was performed using the steepest descent algorithm with and without solute restraints [18]. Then, 1000 ps simulations were performed in NVT and NPT ensembles at temperature 300 K. To control the temperature and pressure during the simulation, the Berendsen thermostats and barostats were applied. Both the relaxed systems were subjected to a final MD run for 100 ns with a time step of 2 fs [47]. For analysis purposes, RMSD, RMSF, *R*_g_, SASA, and hydrogen bonding were recorded. The recorded trajectories were checked for the stability of the HSA–HpzA docked complex in comparison with that of the free state of HSA.

### 3.6. Principal Component Analysis and Free Energy Landscape

Principal component analysis (PCA) is a comprehensive and useful tool to investigate conformational changes of proteins [29]. The theoretical notes on PCA have been explained in various preceding reports [29,31,48]. To realistically discover the variations in the structural configuration of HSA and the HSA–HpzA docked complex, PCA was employed on the equilibrized trajectories from the MD study. The PCA was performed using the GROMACS utilities by calculating the eigenvalues (EVs) and their projection along the two principal components (PC1 and PC2). FEL analysis has been widely used in examining the folding mechanisms and overall stability of protein and protein–ligand complexes [49]. FELs for HSA and the HSA–HpzA docked complex were generated through the *gmx sham* tool of the GROMACS package.

### 3.7. Fluorescence-Based Assay

Fluorescence-based binding was carried out on a Jasco spectrofluorometer FP 6200 (Jasco, Tokyo, Japan). HSA was excited at 280 nm with the emission range set at 300–400 nm. We analyzed the quenching data as per earlier published studies [32,50].

All the experiments were performed in triplicates.

## 4. Conclusions

The possible binding of HpzA with HSA was explored by utilizing molecular docking, MD simulation, PCA, and FEL analyses. Docking results indicated that the binding of HpzA with HSA showed an appreciable binding affinity and many intermolecular interactions. MD trajectory analyses (i.e., RMSD, RMSF, *R_g_*, SASA, and hydrogen bonding) suggested that the HSA–HpzA docked complex was quite stable with minimal conformational alterations. Moreover, PCA and FEL analyses of HSA and HSA–HpzA confirmed that both the systems were stable without any significant change. However, a minor change in the shape and position of the global minima of HSA when bound to HpzA indicated that the HpzA might alter the conformational shape of the binding site of HSA. Fluorescence-based binding ascertained the actual binding affinity between HpzA and HSA, suggesting that HpzA binds to HSA with a significant affinity, validating the in silico observations. Overall, this study reinforces the idea that HSA–HpzA interactions can be explored in AD. The results contribute in many ways to our knowledge and provide a base for setting up an experimental platform of HpzA interactions with HSA to explore them in AD management.

## Figures and Tables

**Table 1:** Binding and thermodynamic parameters obtained for the HSA–HpzA complex from fluorescence quenching studies.

Temperature (°C)	*K_sv_* (10^4^ M^−1^)	*K* 10^5^ M^−1^	*n*	∆*G* kcal mol^−1^	∆*S* cal mol^−1^ K^−1^	∆*H* kcal mol^−1^	*T*∆*S* kcal mol^−1^
15	1.8	0.36	1.07	−7.78	−165.26	−55.38	−47.59
20	3.33	1.17	1.10	−6.96			−48.42
25	5.19	9.35	1.25	−6.13			−49.24

## Data Availability

The data presented in this study are contained within the article and Supplementary Materials.

## Supplementary Materials

The following supporting information can be downloaded online. Figure S1: Molecular structure of Huperzine A: (A) 2D, (B) 3D stick, and (C) 3D ball and stick model of Huperzine A. Figure S2: Different docked conformations of HpzA with HSA. Figure S3: Energy table of different docked conformations of HpzA with HSA.

## References

[1] Fanali G., di Masi A., Trezza V., Marino M., Fasano M., Ascenzi P. (2012). Human serum albumin: From bench to bedside. Mol. Asp. Med..

[2] Rimac H., Debeljak Z., Bojić M., Miller L. (2017). Displacement of Drugs from Human Serum Albumin: From Molecular Interactions to Clinical Significance. Curr. Med. Chem..

[3] He X.M., Carter D.C. (1992). Atomic structure and chemistry of human serum albumin. Nature.

[4] Kragh-Hansen U. (1981). Molecular aspects of ligand binding to serum albumin. Pharmacol. Rev..

[5] Yadav D.K. (2021). Recent Advances on Small Molecule Medicinal Chemistry to Treat Human Diseases-Part III. Curr. Top. Med. Chem..

[6] Shamsi A., Ahmed A., Khan M.S., Al Shahwan M., Husain F.M., Bano B. (2020). Understanding the binding between Rosmarinic acid and serum albumin: In vitro and in silico insight. J. Mol. Liq..

[7] Yadav D.K., Kumar S., Teli M.K., Kim M. (2020). Ligand-based pharmacophore modeling and docking studies on vitamin D receptor inhibitors. J. Cell. Biochem..

[8] Sudlow G., Birkett D.J., Wade D.N. (1976). Further characterization of specific drug binding sites on human serum albumin. Mol. Pharmacol..

[9] Dockal M., Carter D.C., Rüker F. (2000). Conformational Transitions of the Three Recombinant Domains of Human Serum Albumin Depending on pH. J. Biol. Chem..

[10] Fani N., Bordbar A., Ghayeb Y. (2013). Spectroscopic, docking and molecular dynamics simulation studies on the interaction of two Schiff base complexes with human serum albumin. J. Lumin..

[11] Zsila F. (2013). Subdomain IB Is the Third Major Drug Binding Region of Human Serum Albumin: Toward the Three-Sites Model. Mol. Pharm..

[12] Bartus R.T., Dean R.L., Beer B., Lippa A.S. (1982). The cholinergic hypothesis of geriatric memory dysfunction. Science.

[13] Behl C., Moosmann B. (2002). Antioxidant neuroprotection in Alzheimer’s disease as preventive and therapeutic approach. Free Radic. Biol. Med..

[14] Eckert A., Marques C.A., Keil U., Schüssel K., Müller W.E. (2003). Increased apoptotic cell death in sporadic and genetic Alzheimer’s disease. Ann. N. Y. Acad. Sci..

[15] Ma X., Tan C.-H., Zhu D., Gang D.R., Xiao P. (2007). Huperzine A from Huperzia species—An ethnopharmacolgical review. J. Ethnopharmacol..

[16] Wang H., Tang X. (1998). Anticholinesterase effects of huperzine A, E2020, and tacrine in rats. Zhongguo yao li xue bao = Acta Pharmacol. Sin..

[17] Li H., Min Q. (2001). Huperzine A improved the cognition of vascular dementia: A report of 30 patients in therapeutics. Chin. J. Clin. Rehabil..

[18] Yadav D.K., Kumar S., Saloni, Misra S., Yadav L., Teli M., Sharma P., Chaudhary S., Kumar N., Choi E.H. (2018). Molecular insights into the interaction of RONS and Thieno [3, 2-c] pyran analogs with SIRT6/COX-2: A molecular dynamics study. Sci. Rep..

[19] Tiwari M.K., Coghi P., Agrawal P., Shyamlal B.R.K., Jun Yang L., Yadav L., Peng Y., Sharma R., Yadav D.K., Sahal D. (2020). Design, Synthesis, Structure-Activity Relationship and Docking Studies of Novel Functionalized Arylvinyl-1, 2, 4-Trioxanes as Potent Antiplasmodial as well as Anticancer Agents. ChemMedChem.

[20] Shyamlal B.R.K., Yadav L., Tiwari M.K., Mathur M., Prikhodko J.I., Mashevskaya I.V., Yadav D.K., Chaudhary S. (2020). Synthesis, Bioevaluation, Structure-Activity Relationship and Docking Studies of Natural Product Inspired (Z)-3-benzylideneisobenzofuran-1(3H)-ones as Highly Potent antioxidants and Antiplatelet agents. Sci. Rep..

[21] Teli M.K., Kumar S., Yadav D.K., Kim M. (2021). In silico identification of prolyl hydroxylase inhibitor by per-residue energy decomposition-based pharmacophore approach. J. Cell. Biochem..

[22] Mohammad T., Siddiqui S., Shamsi A., Alajmi M.F., Hussain A., Islam A., Ahmad F., Hassan M.I. (2020). Virtual Screening Approach to Identify High-Affinity Inhibitors of Serum and Glucocorticoid-Regulated Kinase 1 among Bioactive Natural Products: Combined Molecular Docking and Simulation Studies. Molecules.

[23] Gadhe C.G., Kim M.-H. (2015). Insights into the binding modes of CC chemokine receptor 4 (CCR4) inhibitors: A combined approach involving homology modelling, docking, and molecular dynamics simulation studies. Mol. Biosyst..

[24] Lobanov M.Y., Bogatyreva N.S., Galzitskaya O.V. (2008). Radius of gyration as an indicator of protein structure compactness. Mol. Biol..

[25] Gadhe C.G., Balupuri A., Cho S.J. (2015). In silico characterization of binding mode of CCR8 inhibitor: Homology modeling, docking and membrane based MD simulation study. J. Biomol. Struct. Dyn..

[26] Ito A., Mukaiyama A., Itoh Y., Nagase H., Thøgersen I.B., Enghild J.J., Sasaguri Y., Mori Y. (1996). Degradation of Interleukin 1β by Matrix Metalloproteinases. J. Biol. Chem..

[27] Hubbard R.E. (2001). Hydrogen Bonds in Proteins: Role and Strength. eLS.

[28] Hubbard R.E., Kamran Haider M. (2010). Hydrogen Bonds in Proteins: Role and Strength. eLS.

[29] Maisuradze G.G., Liwo A., Scheraga H.A. (2009). Principal Component Analysis for Protein Folding Dynamics. J. Mol. Biol..

[30] Fatima S., Mohammad T., Jairajpuri D.S., Rehman T., Hussain A., Samim M., Ahmad F., Alajmi M.F., Hassan I. (2020). Identification and evaluation of glutathione conjugate gamma-l-glutamyl-l-cysteine for improved drug delivery to the brain. J. Biomol. Struct. Dyn..

[31] Altis A., Otten M., Nguyen P.H., Hegger R., Stock G. (2008). Construction of the free energy landscape of biomolecules via dihedral angle principal component analysis. J. Chem. Phys..

[32] Anwar S., Shamsi A., Shahbaaz M., Queen A., Khan P., Hasan G.M., Islam A., Alajmi M.F., Hussain A., Ahmad F. (2020). Rosmarinic Acid Exhibits Anticancer Effects via MARK4 Inhibition. Sci. Rep..

[33] Soares S., Mateus N., de Freitas V. (2007). Interaction of Different Polyphenols with Bovine Serum Albumin (BSA) and Human Salivary α-Amylase (HSA) by Fluorescence Quenching. J. Agric. Food Chem..

[34] Klajnert B., Stanisławska L., Bryszewska M., Pałecz B. (2003). Interactions between PAMAM dendrimers and bovine serum albumin. Biochim. Biophys. Acta (BBA)-Proteins Proteom..

[35] Shamsi A., Al Shahwan M., Ahamad S., Hassan M.I., Ahmad F., Islam A. (2020). Spectroscopic, calorimetric and molecular docking insight into the interaction of Alzheimer’s drug donepezil with human transferrin: Implications of Alzheimer’s drug. J. Biomol. Struct. Dyn..

[36] Waseem R., Anwar S., Khan S., Shamsi A., Hassan I., Anjum F., Shafie A., Islam A., Yadav D.K. (2021). MAP/Microtubule Affinity Regulating Kinase 4 Inhibitory Potential of Irisin: A New Therapeutic Strategy to Combat Cancer and Alzheimer’s Disease. Int. J. Mol. Sci..

[37] Anwar S., Mohammad T., Shamsi A., Queen A., Parveen S., Luqman S., Hasan G.M., Alamry K.A., Azum N., Asiri A.M. (2020). Discovery of Hordenine as a Potential Inhibitor of Pyruvate Dehydrogenase Kinase 3: Implication in Lung Cancer Therapy. Biomedicines.

[38] Anwar S., Shamsi A., Kar R.K., Queen A., Islam A., Ahmad F., Hassan I. (2020). Structural and biochemical investigation of MARK4 inhibitory potential of cholic acid: Towards therapeutic implications in neurodegenerative diseases. Int. J. Biol. Macromol..

[39] Shamsi A., Mohammad T., Anwar S., Nasreen K., Hassan I., Ahmad F., Islam A. (2020). Insight into the binding of PEG-400 with eye protein alpha-crystallin: Multi spectroscopic and computational approach: Possible therapeutics targeting eye diseases. J. Biomol. Struct. Dyn..

[40] Guex N., Peitsch M. (1997). Swiss-Model and the Swiss-Pdb Viewer: An environment for comparative protein modeling. Electrophoresis.

[41] Mohammad T., Mathur Y., Hassan I. (2021). InstaDock: A single-click graphical user interface for molecular docking-based virtual high-throughput screening. Briefings Bioinform..

[42] Trott O., Olson A.J. (2010). AutoDock Vina: Improving the speed and accuracy of docking with a new scoring function, efficient optimization, and multithreading. J. Comput. Chem..

[43] Biovia D.S. (2015). Discovery Studio Modeling Environment.

[44] DeLano W.L. (2002). Pymol: An open-source molecular graphics tool. CCP4 Newsl. Protein Crystallogr..

[45] Van Der Spoel D., Lindahl E., Hess B., Groenhof G., Mark A.E., Berendsen H.J. (2005). GROMACS: Fast, flexible, and free. J. Comput. Chem..

[46] Van Aalten D.M.F., Bywater R., Findlay J.B.C., Hendlich M., Hooft R.W.W., Vriend G. (1996). PRODRG, a program for generating molecular topologies and unique molecular descriptors from coordinates of small molecules. J. Comput. Mol. Des..

[47] Yadav D.K., Kumar S., Choi E.H., Chaudhary S., Kim M.H. (2019). Computational Modeling on Aquaporin-3 as Skin Cancer Target: A Virtual Screening and Molecular Dynamic Simulation Study. Front Chem..

[48] Naqvi A.A.T., Mohammad T., Hasan G.M., Hassan I. (2018). Advancements in Docking and Molecular Dynamics Simulations Towards Ligand-receptor Interactions and Structure-function Relationships. Curr. Top. Med. Chem..

[49] Papaleo E., Mereghetti P., Fantucci P., Grandori R., De Gioia L. (2009). Free-energy landscape, principal component analysis, and structural clustering to identify representative conformations from molecular dynamics simulations: The myoglobin case. J. Mol. Graph. Model..

[50] Shamsi A., Anwar S., Mohammad T., Alajmi M.F., Hussain A., Rehman M.T., Hasan G.M., Islam A., Hassan M.I. (2020). MARK4 inhibited by AChE inhibitors, donepezil and Rivastigmine tartrate: Insights into Alzheimer’s disease therapy. Biomolecules.

