# Supplementary material for: Mechanistic Insight into Binding of Huperzine A with Human Serum Albumin: Computational and Spectroscopic Approaches
Source: Molecules. 2022 Jan 25;27(3):797. doi: 10.3390/molecules27030797 (PMC8839580; doi:10.3390/molecules27030797)
Supplement: Supplementary file 1 [file molecules-27-00797-s001.zip › molecules-1513487-supplementary.pdf]

molecules

# Supplementary Information

## Mechanistic Insight into Binding of Huperzine A with Human Serum Albumin: Computational and Spectroscopic Approaches

**Anas Shamsi [1,2,*], Moyad Shahwan [2,3], Mohd Shahnawaz Khan [4], Fahad A. Alhumaydhi [5], Suliman A. Alsagaby [6], Waleed Al Abdulmonem [7], Bekhzod Abdullaev [8] and Dharmendra Kumar Yadav [9,*]**

[1] Centre for Interdisciplinary Research in Basic Sciences, Jamia Millia Islamia, Jamia Nagar, New Delhi 110025, India
[2] Centre of Medical and Bio-Allied Health Sciences Research, Ajman University, Ajman P.O. Box 346, United Arab Emirates; moyad76@hotmail.com
[3] College of Pharmacy & Health sciences, Ajman University, Ajman P.O. Box 346, United Arab Emirates
[4] Department of Biochemistry, College of Sciences, King Saud University, Riyadh 11451, Saudi Arabia; moskhan@ksu.edu.sa
[5] Department of Medical Laboratories, College of Applied Medical Sciences, Qassim University, Buraydah 52571, Saudi Arabia; f.alhumaydhi@qu.edu.sa
[6] Department of Medical Laboratory Sciences, College of Applied Medical Sciences, Majmaah University, Majmaah 11932, Saudi Arabia; s.alsaqaby@mu.edu.sa
[7] Department of Pathology, College of Medicine, Qassim University, Buraidah 51452, Saudi Arabia; waleedmonem@qumed.edu.sa
[8] Scientific Department, Akfa University, Tashkent 100022, Uzbekistan; b.abdullaev@akfauniversity.org
[9] College of Pharmacy, Gachon University of Medicine and Science, Hambakmoeiro, Yeonsu-gu, Incheon 21924, Korea
* Correspondence: anas.shamsi18@gmail.com (A.S.); dharmendra30oct@gmail.com (D.K.Y.)

molecules

MDPI

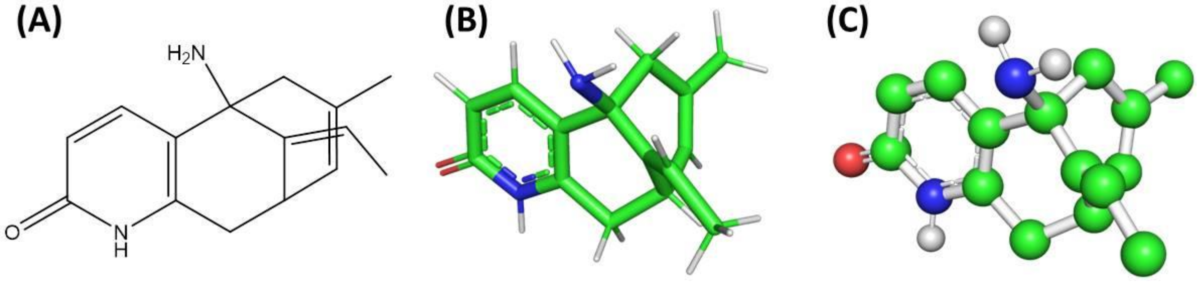


**Figure S1.** Molecular structure of Huperzine A. (A) 2D, (B) 3D stick, and (C) 3D ball and stick model of Huperzine A.

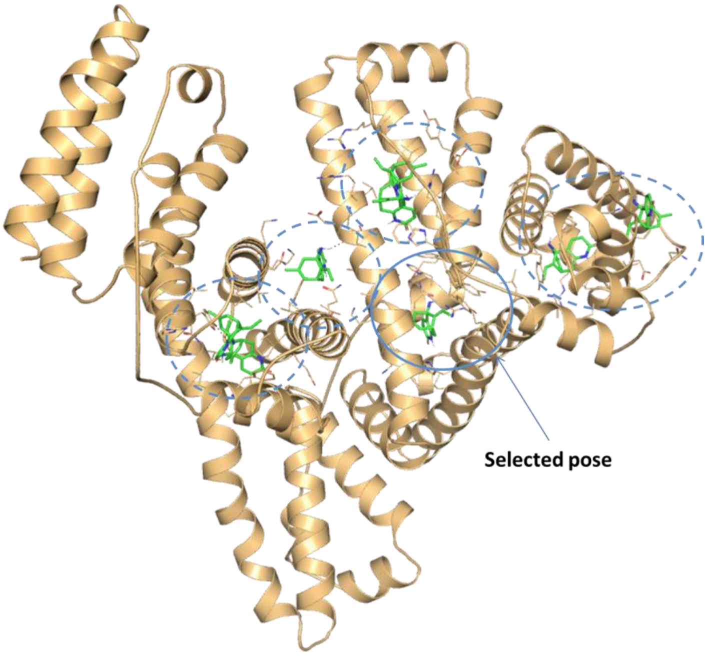


**Figure S2.** Different docked conformations of HpzA with HSA.

Huperzine_A_log - Notepad

File Edit Format View Help

```
# This is an auto-generated log file through InstaDock employing QuickVina-W #
Output will be Huperzine_A_out.pdbqt
Detected 4 CPUs
Reading input ... done.
Setting up the scoring function ... done.
Analyzing the binding site ... done.
Using random seed: -1452432156
Performing search ... done.
Refining results ...
searching finished in 3.277 seconds
done.

mode |   affinity | dist from best mode
     | (kcal/mol) | rmsd l.b.| rmsd u.b.
-----+------------+----------+----------
   1         -7.2      0.000      0.000
   2         -6.5     22.367     24.175
   3         -6.4     52.114     53.432
   4         -6.4     52.192     53.557
   5         -6.4     17.159     19.166
   6         -6.3     18.023     20.214
   7         -6.3     19.911     22.341
   8         -6.2     13.667     15.987
   9         -6.1     13.894     16.503
Writing output ... done.
```

**Figure S3.** Energy table of different docked conformations of HpzA with HSA.
